# Protective effects of paeoniflorin on cardiovascular diseases: A pharmacological and mechanistic overview

**DOI:** 10.3389/fphar.2023.1122969

**Published:** 2023-05-30

**Authors:** Xiaoya Li, Changxin Sun, Jingyi Zhang, Lanqing Hu, Zongliang Yu, Xiaonan Zhang, Zeping Wang, Jiye Chen, Min Wu, Longtao Liu

**Affiliations:** ^1^ Xiyuan Hospital, China Academy of Chinese Medical Sciences, Beijing, China; ^2^ Beijing University of Chinese Medicine, Beijing, China; ^3^ Guang’anmen Hospital, China Academy of Chinese Medical Sciences, Beijing, China

**Keywords:** paeoniflorin, cardiovascular diseases, mechanism, pharmacology, traditional Chinese medicine

## Abstract

**Background and ethnopharmacological relevance:** The morbidity and mortality of cardiovascular diseases (CVDs) are among the highest of all diseases, necessitating the search for effective drugs and the improvement of prognosis for CVD patients. Paeoniflorin (5beta-[(Benzoyloxy)methyl] tetrahydro-5-hydroxy-2-methyl-2,5-methano-1H-3,4-dioxacyclobuta [cd] pentalen-1alpha (2H)-yl-beta-D-glucopyranoside, C_23_H_28_O_11_) is mostly derived from the plants of the family Paeoniaceae (a single genus family) and is known to possess multiple pharmacological properties in the treatment of CVDs, making it a promising agent for the protection of the cardiovascular system.

**Aim of the study:** This review evaluates the pharmacological effects and potential mechanisms of paeoniflorin in the treatment of CVDs, with the aim of advancing its further development and application.

**Methods:** Various relevant literatures were searched in PubMed, ScienceDirect, Google Scholar and Web of Science. All eligible studies were analyzed and summarized in this review.

**Results:** Paeoniflorin is a natural drug with great potential for development, which can protect the cardiovascular system by regulating glucose and lipid metabolism, exerting anti-inflammatory, anti-oxidative stress, and anti-arteriosclerotic activities, improving cardiac function, and inhibiting cardiac remodeling. However, paeoniflorin was found to have low bioavailability, and its toxicology and safety must be further studied and analyzed, and clinical studies related to it must be carried out.

**Conclusion:** Before paeoniflorin can be used as an effective therapeutic drug for CVDs, further in-depth experimental research, clinical trials, and structural modifications or development of new preparations are required.

## 1 Introduction

Cardiovascular diseases (CVDs), a chronic non-communicable disease, refer to a group of disorders of the heart or blood vessels. Common CVD types include coronary heart disease, aortic disease, peripheral arterial disease, and stroke (Olvera Lopez et al., 2022). Ischemic heart disease and ischemic stroke are collectively referred to as atherosclerotic cardiovascular disease (ASCVD), which is the most prevalently encountered CVD (Arnett et al., 2019; Wong et al., 2022). Several risk factors for CVDs have been identified, such as dyslipidemia, diabetes, metabolic syndrome, hypertension, chronic kidney disease, smoking, age, and genetic history (Yusuf et al., 2020; O'Sullivan et al., 2022). At present, the mainstream therapy of CVDs is medication, including antiplatelet drugs, anticoagulants, statins, anti-thrombotic drugs, beta receptor blockers, antiarrhythmic agent and nitrates (Leong et al., 2017). Despite the availability of a wide range of drugs for clinical use, the morbidity and mortality of CVDs are the highest among all diseases, which not only pose a severe challenge to human health but also bring a huge economic burden to individuals, families, and society (Kalogeropoulos and Butler, 2022; Townsend et al., 2022). As an important problem that must be faced and solved, it is pivotal to seek effective drugs for treating CVDs, and improve the prognosis and quality of life of CVDs patients.

**FIGURE 1:**
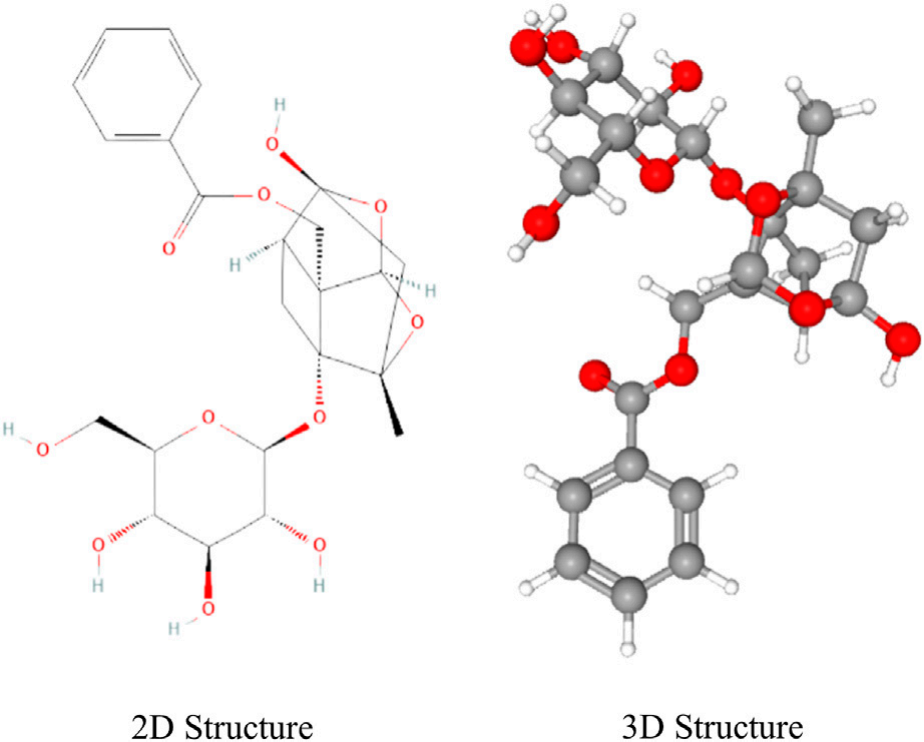
Chemical structure of paeoniflorin.

Paeoniflorin (C_23_H_28_O_11_, PubChem CID: 442534), with its chemical name as 5beta-[(Benzoyloxy)methyl] tetrahydro-5-hydroxy-2-methyl-2,5-methano-1H-3,4-dioxacyclobuta [cd] pentalen-1alpha (2H)-yl-beta-D-glucopyranoside, is a pinane monoterpene bitter glucoside distributed in the roots of Paeonia albiflora Pall, P. suffrsticosa Andr, P. delarayi Franch and other Paeoniaceae (Paeonia L.). Paeoniflorin is mostly derived from plants of Paeoniaceae (a single genus family). In 1963, paeoniflorin was first isolated from the roots of Paeonia albiflora and named by Shibata and Nakahara (1963). Further studies showed that the basic skeleton of paeoniflorin is a pinane derivative, which is chemically stable and is a water-soluble monoterpene glycoside (Xia et al., 2007; Sun B et al., 2017). As shown in Figure 1, β-d-glucopyranosyl, benzoyl, and semi-ketal-acetal structures are linked to the backbone, which formed the complete chemical structure of paeoniflorin (Zhang et al., 2022).

**FIGURE 2:**
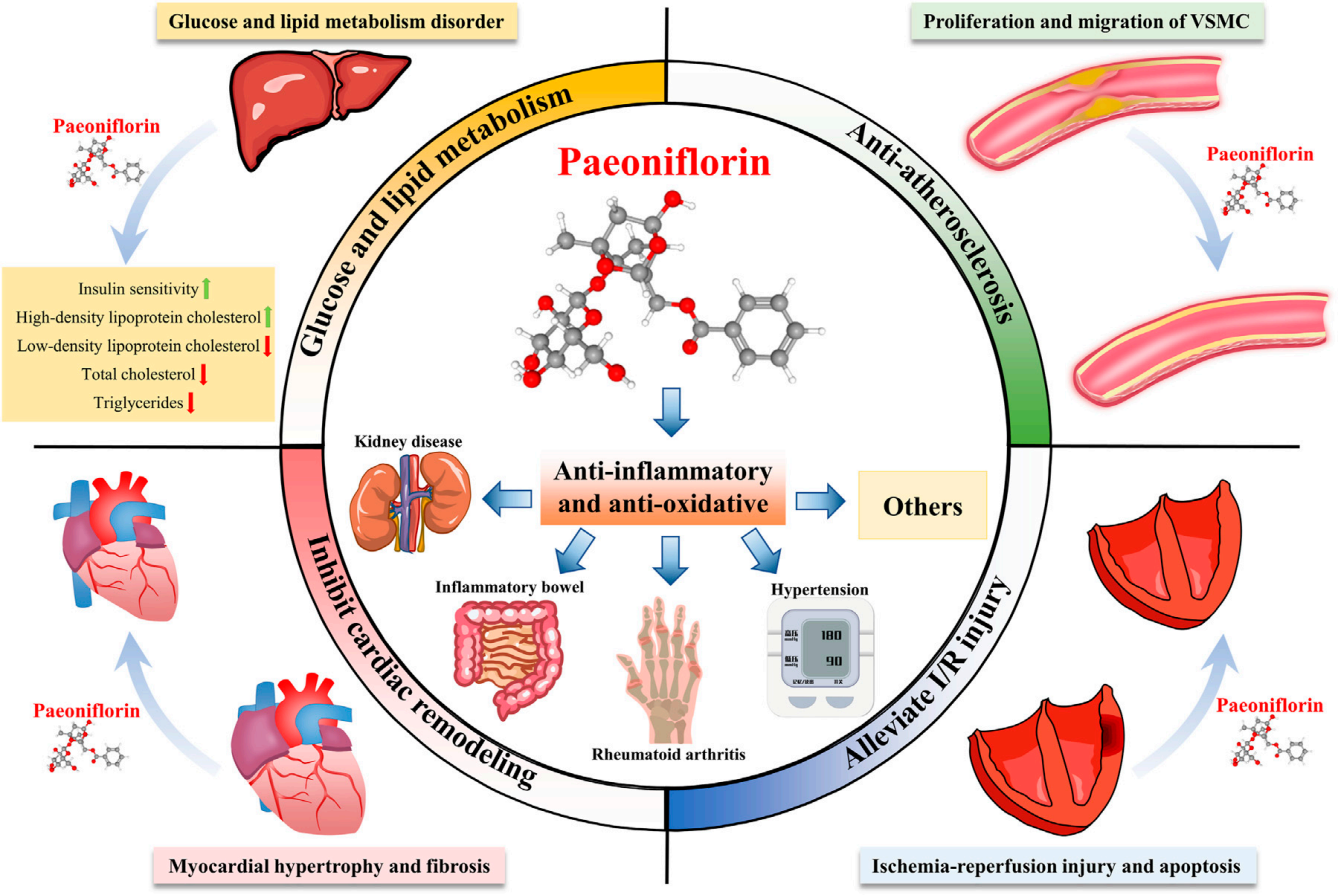
Multi-system pharmacological effects of paeoniflorin. Paeoniflorin has therapeutic effects on many diseases, and can prevent and treat kidney diseases, inflammatory bowel disease, rheumatoid arthritis, hypertension and other diseases through anti-inflammatory and anti-oxidant effects. In addition, paeoniflorin can improve glucose and lipid metabolism, resist atherosclerosis, inhibit myocardial remodeling, and improve ischemia-reperfusion injury.

Studies have shown that the paeoniflorin can regulate glucose and lipid metabolism, exert anti-inflammatory, anti-oxidative stress, and anti-arteriosclerotic activities, improve cardiac function, and inhibit cardiac remodeling, thus making it a promising candidate for the treatment of CVDs and protection of the cardiovascular system (Figure 1). Whilst several previous reviews have approached the usage of paeoniflorin for neurological disorders and neurodegeneration (Jiao et al., 2021; Hong et al., 2022), analgesia (Lin et al., 2019; Ruan et al., 2021), antidepressants (Zhang et al., 2021a; Lei et al., 2022), neuroprotection (Chen et al., 2020; Guo et al., 2021), and immunomodulation (Chen et al., 2016; Yang and Wei, 2020), a review specific to paeoniflorin’s protective effect on the cardiovascular system is notably lacking. At present, no review articles about paeoniflorin protecting the cardiovascular system have been found in PubMed or other relevant databases. This article is thus dedicated to providing an overview of the pharmacological effects and possible mechanisms of paeoniflorin in the treatment of CVDs, in the hopes of further advancing its development and application (Figure 2).

## 2 Plant sources of paeoniflorin

Paeoniaceae is a monofamily consisting of 34 species, mainly distributed in temperate Eurasia, northwest Africa and western North Americ (Hong, 2011). In China, plants of the Paeoniaceae family are widely cultivated and used for their special medicinal benefits and ornamental value, which makes China the region with the highest concentration of Paeoniaceae in the world (Zhou et al., 2021). The most representative plant in Paeoniaceae is Paeonia lactiflora Pall (Shaoyao in Chinese). As a medicinal plant, Shaoyao was first recorded in the book Shennong’s Herbal Classic of Materia Medica and has a history of more than 2000 years (Xu et al., 2021). In Zhang Zhongjing’s book “Treatise on Febrile Diseases,” there are 113 prescriptions, 33 of which contain Shaoyao, accounting for 29% of all prescriptions. In the northern and southern dynasties, Tao Hongjing pointed out that Shaoyao can be divided into Chishao (Paeoniae Radix Rubra) and Baishao (Paeoniae Radix Alba) in Chinese, which are included in modern Chinese pharmacopoeia (Committee, 2020).

As the traditional Chinese medicines in China, Chishao and Baishao are all processed from the roots of Paeoniaceae plants. The Chinese Pharmacopoeia records that Chishao is the dried root of Paeonia lactiflora Pall or Paeonia veitchii Lynch, while Baishao is the dried root of Paeonia lactiflora Pall (Committee, 2020). Paeoniflorin is the main active ingredient of Chishao and Baishao (Zhang et al., 2022). Chishao and Baishao derive from the same species or closely related plants, which contain similar chemical components, but the paeoniflorin content in Chishao is slightly higher than that in Baishao. According to the Chinese Pharmacopoeia, the paeoniflorin content in dried Baishao should be higher than 1.6%, and in Chishao should be higher than 1.8% (Committee, 2020). In addition to the roots of Paeoniaceae, paeoniflorin is distributed throughout the plant including flowers, stems, leaves, fruits, seeds and rhizomes (Zhang et al., 2022). Since the biosynthetic pathway of paeoniflorin has not been fully elucidated, the chemical synthesis process is complicated and the production cost is high, therefore, the currently applied paeoniflorin is mainly extracted from Paeoniaceae plants (SUN L C et al., 2017). However, the low yield of this generation method does not allow for economical mass production and is increasingly difficult to meet the current increasing demand in pharmaceutical formulations. In the next stage, we should further study the specific modification stage of paeoniflorin in structure and function, further clarify the biosynthesis pathway, and increase the production of paeoniflorin on the basis of environmental protection.

## 3 Pharmacological effects of paeoniflorin on the cardiovascular system

### 3.1 Regulation of glucose and lipid metabolism

Dyslipidemia, obesity, and overweight are risk factors for hypertension, coronary heart disease, and peripheral vascular disease (Seravalle and Grassi, 2017; Pirillo et al., 2021). Paeoniflorin has been shown to reduce body weight, regulate lipid metabolism and serum glucose levels, increase insulin sensitivity in obese mice, and improve the accumulation of ectopic lipids (Zhang et al., 2015; Ma et al., 2017a). It has also been reported to significantly reduce the levels of total cholesterol, low-density lipoprotein, and triglycerides in hyperlipidemic rats (Yang et al., 2004; Li et al., 2017a). However, other studies suggest that the effect of paeoniflorin is mainly achieved by lowering cholesterol, with only a limited impact on triglyceride levels (Zhang et al., 2015). Thus, further investigation and discussion on the effects of paeoniflorin on triglyceride metabolism is warranted. The identification of lipid metabolism genes targeted by paeoniflorin has indicated it can regulate lipid synthesis and metabolism through several signaling pathways, such as the *de novo*, lipid oxidation, cholesterol synthesis and output pathways (Zhang et al., 2015). It has been suggested that paeoniflorin can reduce lipid synthesis by inhibiting SREBP-1c via the *de novo* pathway and decreasing the expression of FAS, ACC-α, and other proteins (Ma et al., 2017a). It has been found that enzyme 3-hydroxy-3-methylglutaryl coenzyme A reductase (HMG-COAR) is an important enzyme for cholesterol synthesis, and cytochrome P4507A1 (CYP7A1) is a rate-limiting enzyme in the classical pathway of bile acid synthesis. The two enzymes are the key enzymes to regulate the metabolic balance of cholesterol in the body (Osaki et al., 2015). Studies have shown that paeoniflorin can regulate cholesterol synthesis and metabolism by decreasing HMG-CoAR activity and increasing CYP7A1 expression (Zhang et al., 2015; Ma et al., 2017a). Furthermore, Paeoniflorin can attenuate the lipolysis in adipocytes and ameliorate tumor necrosis factor-α (TNF-α) -induced dysfunction of adipocytes (Kong et al., 2013). In addition, the N-acetylgalactosamine transferase 2-angiopoietin like protein 3-lipoprotein lipase (GALNT2-ANGPTL3-LPL) pathway, which is closely related to dyslipidemia and able to directly affect HDL metabolism, has been reported to be effectively regulated by paeoniflorin (Khetarpal et al., 2016; Xiao et al., 2017). A study by Xiao and colleagues showed that 6-week paeoniflorin treatment was able to significantly reduce ANGPTL3 expression, promote GALNT2 and LPL expression, increase serum HDL cholesterol levels, and regulate lipid metabolism in ApoE (−/−) mice (Xiao et al., 2018) (Table 1).

**TABLE 1:** Summary of pharmacological effects of paeoniflorin (PF).

Type	Model	Dose (PF)	Group	Duration	Method	Effect (PF group)	References
vitro	LPS-exposed microglia model	48 μg/mL (100 μM), 96 μg/mL (200 μM)	(1) Control (intact cells); (2) An LPS-treated group (100 ng/mL); (3) Low-dose group (LPS + PF 100 μM); (4) High-dose group (LPS + PF 200 μM).	1 h	ELISA kits, Western blotting analyses, qRT-PCR.	NF-κB↓, TNF-α↓, IL-1β↓, IL-6↓, IFNγ↓, IL-4↑, IL-10↑, ROS↓, MOD↓, SOD↑, GSH↑	Chen et al. (2020)
vivo	Non-alcoholic fatty liver disease model (HFD-fed C57BL/6J mice)	Corresponding diet supplemented with 0.05% PF	(1) Control (normal control mice); (2) Control + PF; (3) High-fat diet-fed mice; (4) High-fat diet-fed mice + PF.	24 weeks	Biochemical analysis, histologcal analysis, qRT-PCR.	TC↓, TG↓, LDL-C↓, HDL-C↓, ALT↓, AST↓, FAS↓, PPARα ↑, HMGCR↓, PPARγ↓, ABCA-1↓, TNF-α↓, IL-1↓, IL-6↓, MCP-1↓	Zhang et al. (2015)
vivo	Non-alcoholic fatty liver disease model (HFD-fed male Sprague-Dawley rats)	20 mg/kg	(1) Control; (2) HFD group; (3) HFD + PF (PF 20 mg/kg).	4 weeks	Biochemical analysis, histologcal analysis, Western blotting analyses.	TC↓, TG↓, ALT↓, AST↓, SREBP-1c↓, FAS↓, ACCα↓, HMGCR↓, CYP7A1↑, p-IRS-1↓, p-Akt↑, p-GSK3β↑, ROS↓, MOD↓, CYP2E1↓, HOMA-IR index↓	Ma et al. (2017a)
vivo + vitro	Atherosclerosis rat model was established by administration of excessive vitamin D and cholesterol. Cell model of atherosclerosis (VSMCs)	vivo: 10, 20 mg/kg;	Vivo: (1) Control (SD rats + saline); (2) Atherosclerotic group (vitamin D3 + fat emulsion); (3) Low-dose group (PF 10 mg/kg/day); (4) High-dose group (PF 20 mg/kg/day); (5) Simvastatin group (simvastatin 5 mg/kg).	vivo: 15 weeks;	Histopathologic evaluation of aortas, MTT assay, ELISA kits, qRT-PCR, Western blot analysis.	TLR4↓, MyD88↓, IκBα↓, NF-κB↓, TC↓, TG↓, LDL-C↓, IL-1β↓, TNF-α↓, IL-6↓	Li et al. (2017a)
		vitro: 5, 10, 30, 60, 100 μmol/L	vitro: (1) Control; (2) Atherosclerotic group (palmitic acid 100 μmol/L); (3) Low-dose group (PF 60 μmol/L); (4) High-dose group (PF 100 μmol/L).	vitro:1 h			
vitro	3T3-L1 adipocyte insulin resistance model	12.5, 25, 50, 100 mg/L	(1) Control; (2) TNFα group (10 ng/mL); (3) Insulin group (10 nM); (4) PF group; (5) PF + TNFα group; (6) TNFα + insulin group.	24 h	ELISA kits, BCA protein assay, MTT assay, Western blot analysis, qRT-PCR.	TNF-α↓, PPARγ↓, IL-6↓, MCP-1↓	Kong et al. (2013)
vivo	Apolipoprotein E null mice	10, 20, 30 mg/kg	(1) Control (C57BL/6J); (2) Untreated ApoE −/− group; (3) PF + ApoE −/−group (10, 20, 30 mg/kg).	6 weeks	T-AOC detection kit, qRT-PCR, Western blot analysis.	ANGPTL3↓, GALNT2↑, LPL↑, TC↓, LDL-C↓, TG↓, HDL-C↑	Xiao et al. (2017)
vivo + vitro	Experimental DIC mouse model; cell inflammation model (RAW 264.7 murine macrophages)	15, 30, 60 mg/kg	vivo: (1) Control; (2) LPS (60 mg/kg); (3) Heparin (10 IU/kg); (4) low-PF treatment (15 mg/kg); (5) Medium-PF treatment (30 mg/kg); (6) High-PF treatment (60 mg/kg). Thereafter mice in each group were randomly divided into three groups: 0-h group, 2 h group, and 8 h group.	vivo: every 2 h	MTT assay, ELISA kits, Western blot analysis.	NF-κB↓, TNF-α↓, IL-6↓, TLR4↓, IκBα↓	Fang et al. (2020)
			vitro: (1) Control, (2) LPS (10 μg/mL), (3) PF treatment groups (30, 60, 120 μm).	vitro: 30 min			
vitro	Coculture of differentiated 3T3-L1 adipocytes and RAW 264.7 macrophages	6.25, 12.5, 25, 50, 100 mg/L	(1) Vehicle (0.1% DMSO); (2) Negative control (TNF α or MCP-1 levels released by macrophages alone, FFA from adipocytes alone); (3) PF treatment (1–100 mg/L).	24 h	MTT assay, ELISA kits, acyl-coenzyme A oxidase-based colorimetric assay kit, BCA protein assay, Western blot analysis, qRT-PCR.	MAPKs↓, NF-κB↓, ERK1/2↓, p38↓, JNK↓, IKK↓, TNF-α↓, MCP-1↓, FFA↓	Jiang et al. (2012)
vitro	Lysophosphatidylcholine-induced inflammatory factor production in HUVECs	1, 10, 100 μmol/L	(1) Control; (2) LPC, (1 μg/mL); (3) PF + LPC group (PF: 1, 10, 100 μmol/L); (4) PF group (100 μmol/L); (5) ethanol (less than 0.1% v/v).	2 h	MTT assay, ELISA kits, qRT-PCR, Western blot analysis.	HMGB1↓, RAGE↓, TLR-2↓, TLR-4↓, NF-κB↓, ICAM-1↓, MCP-1↓, IL-6↓, TNF-α↓	Li et al. (2013)
vitro	LPS-stimulated RAW264.7 macrophages	25, 50, 100, 200, 400 μM	(1) Control; (2) LPS (1 μg/mL); (3) PF + LPS group. (PF: 100, 200, 400 μg/mL)	24 h	ELISA kits, Western blot analysis, qRT-PCR.	NF-κB↓, ERK1/2↓, MAPKs↓, COX-2↓, iNOS↓, ROS↓	Li et al. (2022)
vitro	LPS-stimulated RAW264.7 macrophages	11, 33, 100 µM	(1) Control; (2) LPS (60 mg/kg); (3) PF + LPS group (PF: 11, 33, 100 μM); (4) DXM + LPS group (DXM: 33 μM).	2 h	Microplate reader, ELISA kits.	NO↓, TNF-α↓, IL-6↓	Bi et al. (2017)
vitro	M1/M2 cells differentiated from bone marrow progenitor cells of male Balb/c mice	1, 10, 100 μg/mL	(1) Control; (2) LPS (100 ng/mL); (3) PF + LPS group (PF: 1, 10, 100 μg/mL); (4) IL-4 group (20 ng/mL); (5) IL-4 + PF group (PF: 1, 10, 100 μg/mL).	24 h	CCK-8, ELISA kits, Western blot analysis, qRT-PCR, NO assay kit, arginase assay Kit, immunofluorescence analysis.	NF-κB↓, iNOS↓, NO↓, STAT6↑, IL-4↑, M1↓, M2↑	Zhai et al. (2016)
vivo + vitro	Male C57/BL6 mice, RAW264.7 macrophages	vivo: 1, 5, 25 mg/kg	vivo: (1) Control; (2) LPS (200 ng); (3) PF + LPS group (PF: 1, 5, 25 mg/kg).	vivo: 1 week	MTT assay, ELISA kits, qRT-PCR, Western blot analysis, calcium imaging, protein kinase C activity assay kit.	TNF-α↓, IL-1β↓, IL-33↓, NF-κB↓, TLR4↓, MAPKs↓, IκBα↓, Ca2+ influx↓	Li et al. (2020)
		vitro: 0–25 μM	vitro: (1) Control; (2) LPS (1 μg/mL); (3) PF group (10 μg/mL) (4) PF + LPS group.	vitro: 24 h			
vivo + vitro	vivo: Diabetic mice model (8–10 weeks males WT- C57BL/6J and TLR4−/− mice);	vivo: 25, 50, 100 mg/kg	vivo: (1) WT; (2) WT + STZ; (3) WT + STZ + PF (PF:25, 50, 100 mg/kg); (4) TLR4−/− mice; (5) TLR4−/− + STZ.	vivo: 12 weeks	Pathology and immunohistochemistry analysis, CCK-8 kit, cell migration assay, flow cytometry analyses, confocal microscopy analysis, ELISA kits, Western blotting analyses, qRT-PCR.	TLR4↓, IL-1β↓, MCP-1↓, iNOS↓, MyD88↓, IκBα↓, NF-κB↓, p-IRAK1↓, Trif ↓, p-IRF3↓, TNF-α↓, IL-1β↓, MCP-1↓	Shao et al. (2019)
	vitro: BMDM (6–8 weeks old male TLR4−/− and C57BL/6JWT).	vitro: 10^−8^–10^−3^ mol/L	vitro: (1) Normal glucose concentration control group (LG), (2) Normal glucose concentration + PF intervention group (LG + PF), (3) High-glucose stimulation group (HG), (4) PF intervention group (HG + PF), (5) Normal glucose concentration TLR4 knockout group (TLR4−/−), (6) TLR4 knockout macrophages + high-glucose stimulation group (TLR4−/− + HG), and (7) TLR4 knockout macrophages + high-glucose stimulation + PF intervention group (TLR4−/− + HG + PF).	vitro: 24 h			
vitro	LPS-stimulated HUVECs	20, 50, 80 µM	(1) Control; (2) LPS (1 μg/mL); (3) PF group (PF: 20, 50, 80 µM) (4) PF + LPS + 4-PBA (PF: 20, 50, 80 μM; PBA: 5 mM).	24 h	MTT assay, ELISA kits, qRT-PCR, Western blot analysis, transmission electron microscope assay, immunofluorescence staining.	IL-6↓, MCP-1↓, GRP78↓, CCAAT↓, IRE1α↓, NF-κB↓	Chen et al. (2018a)
vivo + vitro	vivo: A mouse model of cutaneous Arthus reaction;	vivo: 25, 50 mg/kg	vivo: (1) Control; (2) IC (IgG 40 µg/30 µL in PBS); (3) PF + IC group (PF: 25, 50 mg/kg);	vivo: 0.5 h	Immunohistochemistry, analysis of myeloperoxidase activity, ELISA kits, Western blotting analyses, qRT-PCR, adhesion assay.	E-selectin↓, ICAM-1↓, TNF-α↓, p38↓, JNK↓	Chen et al. (2013)
	vitro: TNF-α-induced HDMECs	vitro: 125, 250, 500 μM	vitro: (1) Control; (2) TNF-α(10 ng/mL); (3) PF + TNF-α group (PF: 125, 250, 500 μM).	vitro: 0.5 h			
vivo + vitro + vivo	vivo: ANIT-induced cholestatic liver injury model (C57BL/6 mice)	vivo: 75, 150, 300 mg/kg	vivo: (1) Control; (2) ANIT (80 mg/kg); (3) PF + ANIT (PF 75, 150, 300 mg/kg); (4) Red Tuihuang particles (3.9 g/kg) + ANIT.	vivo: 10 days	Uridine diphospho-glucuronosyltransferase assay, MDA assay kit, Western blotting analyses, MTT assay.	ALT↓, AST↓, TBIL↓, DBIL↓, TBA↓, ALP↓, MDA↓, GSH↑, Nrf2↑, Ntcp↑, Nox4↓, NTCP↑, NOX4↓, NQO1↑	Mao et al. (2022)
	vitro: Nrf2 plasmid or siRNA-Nrf2 transfection on LO2 cells	vitro: 4, 20, 100, 500 µM	vitro: (1) Control; (2) ANIT (50 µM); (3) PF + ANIT (PF 100 µM)	vitro: 24 h			
	vivo: ANIT-induced cholestatic liver injury model (Nrf2^−/−^ mice)		vivo: (1) Control; (2) ANIT (80 mg/kg); (3) PF + ANIT (PF 300 mg/kg).	vivo: 10 days			
vivo	SAP lung injury rat model	40 mg/kg	(1) Sham operation group; (2) SAP group (5% sodium taurocholate (1 mL/kg) was retrogradely injected into the biliopancreatic duct at a rate of 0.1 mL/min); (3) PF treatment group (40 mg/kg); (4) Dexamethasone-positive control group (2 mg/kg).	24 h	H&E Staining, biochemical indicators, ELISA kits, Western blotting analyses.	AMY↓, lipase activity↓, LDH↓, MDA↓, SOD↑, TNF-α↓, IL-6↓, IL-10↑, Cyt-Nrf2↑, HO-1↑, NQO1↑	Hu and Yang (2022)
vivo	A hyperlipidemic rat model	500 mg/kg, 300 mg/kg, and 100 mg/kg	(1) Normal control group; (2) High cholesterol group; (3) High cholesterol + simvastatin group; (4) High cholesterol + PF group (PF: 500 mg/kg, 300 mg/kg, 100 mg/kg).	12 weeks	Rat liver histology and immunohistochemical analysis, Western blotting analyses.	HMG-CoAR↓, LDLR↑, PPAR-α↑, CYP7A1↑, SOD↑, MDA↓, Nrf2↑	Hu et al. (2017)
vitro	Oxidative damage model induced by advanced oxidation protein products (AOPPs) in HUVECs	50–200 μM	(1) Control; (2) BSA (200 μg/mL); (3) AOPPs (200 μg/mL); (4) PF (200 μM); (5) Different inhibitors (RAGE blocking agent FPS-ZM1, NADPH oxidase inhibitor Apocynin, ROS scavenger NAC, NF-κB inhibitor BAY11-7082).	1 h	MTT assay, DCFH-DA staining, flow cytometry, confocal microscopy, ATP determination kit, Western blotting analyses.	MMP↑, ATPz↑, NF-κB p65↓, Nox1↓, Nox2↓, HIF-4α↓, VEGF↓, RAGE↑	Song et al. (2017)
vitro	DOX-induced cardiomyocyte apoptosis model (H9c2 cell)	100 μmol/L	(1) Control (cultured in normal condition); (2) DOX group (incubated with 5 μmol/L DOX for 24 h); (3) PF + DOX group (cells were treated with 100 μmol/L PF for 2 h prior to exposure to 5 μmol/L DOX for 24 h); (4) PF group (incubated with 100 μmol/L PF for 26 h).	26 h	MTT assay, cardiomyocyte apoptosis assay, intracellular ROS assay, Western blotting analyses, qRT-PCR.	ROS↓, microRNA-1↓, Bcl-2↑	Li et al. (2016)

Abbreviations: AMY, serum amylas; ANIT, α-naphthalene isothiocya-nate; AOPPs, advanced oxidation protein products; ApoE −/−, apolipoprotein E null; ALT, alanine aminotransferase; ALP, alkaline phosphatase; AST, aspartate aminotransferase; ATP, adenosine triphosphate; BMDMs, bone marrow-derived macrophages; BSA, bovine serum albumin; CCK-8, cell counting kit-8; COX, cyclooxygenase; DBIL, direct bilirubin; DCFH-DA. 2′, 7′-dichlorofluorescein-diacetate; DOX, doxorubicin; DXM, dexamethasone; ELISA, enzyme-linked immunosorbent assay; ERK, extracellular signal-regulated kinase; FFA, free fatty acid; HDL-C, high-density lipoprotein cholesterol; HDMECs, human dermal microvascular endothelial cells; HFD, high-fat diet; HMG-CoAR, 3-hydroxy-3-methylglutharyl-coenzyme A reductase; HO-1, heme oxygenase-1; HOMA-IR, homeostasis model of insulin resistance; HUVECs, human umbilical vein endothelial cells; IC, immune complex; iNOS, inducible nitric oxide synthase; ICAM, inter cellular adhesion molecule; IL, interleukin; JNK, c-Jun N-terminal kinase; LDL-C, low-density lipoprotein cholesterol; LPC, lysophosphatidylcholine; LPS, lipopolysaccharide; MAPK, mitogen-activated protein kinase; MMP, matrix metalloproteinase; MTT, thiazolyl blue tetrazolium bromide; NF-E2 p45-related factor 2 (Nrf2); NF-κB, nuclear factor-kappa B; Nqo1 (NRF2 downstream gene); NTCP, sodium taurocholate co-transporting polypeptide; PBA, phenylbutric acid; ROS, reactive oxygen species; RT-qPCR, reverse transcription polymerase chain reaction; SAP, severe acute pancreatitis; SOD, superoxide dismutase; STZ, streptozotocin; TBA, total bile acid; TBIL, total bilirubin; TC, total cholesterol; TG, triglycerides; TLR, Toll-like receptor; TNF, tumor necrosis factor; TPG, total paeony glucosides VEGF, vascular endothelial growth factor; WT, Wild-type.

↑ represents upregulation of expression andid ↓ represents downregulation of expression.

### 3.2 Anti-inflammatory effect

Inflammation is a major pathological factor contributing in the occurrence and progression of CVDs such as atherosclerosis, thrombosis, myocardial infarction, and ischemia-reperfusion injury (Kim and Conte, 2020). Paeoniflorin has prominent anti-inflammatory effects, which can act by regulating a variety of signalling pathways, such as the GPCR, MAPKs/NF-κB, PI3K/Akt/mTOR, JAK2/STAT3, and TGFβ/Smad pathways (Tu et al., 2019; Xin et al., 2019; Zhang and Wei, 2020). Paeoniflorin can achieve the regulation of anti-inflammatory effects on macrophages and endothelial cell dysfunction by regulating upstream and downstream molecules of NF-κB signaling pathway, which may indicate that paeoniflorin can treat and alleviate cardiovascular diseases from anti-inflammatory mechanism (Table 1).

TNF-α, a prototype member of the tumor necrosis factor superfamily, is predominantly secreted by macrophages and monocytes. Being a major proinflammatory cytokine, it triggers a series of inflammatory processes (Aggarwal et al., 2012). Paeoniflorin can attenuate TNF-α expression by suppressing the activation of the NF-κB signaling pathway (Fang et al., 2020). The paracrine loops of free fatty acids (FFA) and TNF-α present between adipocytes and macrophages form a vicious cycle of inflammation that increases inflammatory changes and insulin resistance in adipose tissue of obese individuals (Suganami et al., 2005). Meanwhile, paeoniflorin is capable of lowering FFA and TNF-α levels by interfering in the interaction between adipocytes and macrophages, thereby impeding the occurring of related inflammatory reactions (Jiang et al., 2012). Research has also demonstrated its capacity to inhibit TNF-α-stimulated phosphorylation at ERK, JNK, and IKK subunits, as well as reduce the expression of pro-inflammatory factors such as IL-6 and MCP-1 in adipocytes (Kong et al., 2013). Pathogen pattern recognition receptors, such as Toll-like receptors (TLRs), can regulate the cytokine response to various inflammatory stimuli (de Kleijn and Pasterkamp, 2003). TLR4 can mediate the activation of the downstream factors MyD88 and NF-κB and induce a surge in proinflammatory cytokines like Il-1β, IL-6, and TNF-α (Fitzgerald et al., 2001). Moreover, paeoniflorin has been found to effectively regulate TLR-2 and TLR-4 expression, thereby decreasing inflammation by suppression of the TLR4/MyD88/NF κB pathway (Li et al., 2013; Li et al., 2017a) (Table 1).

Macrophages are key effectors of inflammation and the innate immune response, and play an pivotal role in the pathogenesis of many CVDs (Xu et al., 2022). The study found that paeoniflorin can reduce the inflammatory response of LPS-stimulated RAW264.7 macrophages by inhibiting the NF-κB/ERK1/2/p38 MAPK signaling pathway (Bi et al., 2017; Li et al., 2022). Linked to that, macrophages show the capability to polarize to M1 and M2 phenotypes (Murray, 2017). Paeoniflorin can decrease the pro-inflammatory activities of M1 macrophages by downregulating inducible nitric oxide synthase (iNOS) expression and NO production through the NF-κB signaling pathway, while simultaneously facilitating the anti-inflammatory function of M2 macrophages by upregulating Arg-1 activity attainable by modulation of the IL-4/STAT6 signaling pathway (Zhai et al., 2016). In addition, Interleukin 33 (IL-33) is a newly identified member of the interleukin family that macrophages, such as M2 macrophages, can secrete (Furukawa et al., 2017). When tissue or cell injury occurs, the release of IL-33 increases, and further adjusts macrophage function by controlling chemokine expression and triggering macrophage polarization (Joshi et al., 2010). Paeoniflorin can regulate macrophage polarization and inhibit IL-33 production by macrophages by regulating the TLR4/NF-κB/P38 MAPK signaling pathway (Chen et al., 2020; Li et al., 2020). However, other studies have suggested that paeoniflorin cannot directly inhibit the activation of macrophages but affects macrophages by inhibiting the expression of iNOS and the production of TNF-α, IL-1β, and MCP-1 (Shao et al., 2019). Therefore, the specific action mode of paeoniflorin in regulating the function of macrophages deserves further study and discussion (Table 1).

Endothelial dysfunction is the primary pathological manifestation of several CVDs (Badimon et al., 2012). On a molecular level, inflammation associated with endoplasmic reticulum (ER) stress appears to be the primary culprit of endothelial dysfunction (ED) (Battson et al., 2017). Paeoniflorin has been reported to be able to restrain the inositol enzyme 1alpha (IRE1α)/NF-κB pathway, eventually diminishing vascular inflammation related to endoplasmic reticulum stress and subsequently reducing endothelial dysfunction (Chen et al., 2018a). Additionally, endothelial cell injury can lead to increased inter cellular adhesion molecule-1 (ICAM-1) expression, which further induces monocyte migration, adhesion, activation, and the ensuing intensify of inflammatory response locally (Most et al., 1992; Frank and Lisanti, 2008). Studies have unveiled that paeoniflorin has the capacity to reduce ICAM-1 expression and inhibit vascular damage (Chen et al., 2013) (Table 1).

By combing through the above research literature, we found that currently, there is limited evidence to directly verify the anti-inflammatory role of paeoniflorin in cardiovascular disease. However, the results of the *in vitro* studies do demonstrate a remarkable anti-inflammatory effect for paeoniflorin, which can regulate macrophages and endothelial cells, the two vital cell types in the cardiovascular system. Therefore, its potential anti-inflammatory role in cardiovascular disease warrants further exploration and examination. Notwithstanding, there are a few shortcomings visible in the above research. For example, the highest dose of paeoniflorin used by studies conducting cell experiments stands in great disparity, with some suggesting a maximum dose of 100 μM (Bi et al., 2017), while others pointing out that 15 μM paeoniflorin could show obvious cytotoxicity when cultured for 48 h (Li et al., 2020). Since dose concentration is the essential information in pharmacology, it may significantly hamper forward fundamental and clinical studies and, therefore, subsequent researches should focus on precisely figuring out the maximum efficacious dose of paeoniflorin *in vivo* or *in vitro* studies and provide detailed, experimental data for further studies (Table 1).

### 3.3 Anti-oxidative effect

In addition to hypertension, diabetes, dyslipidemia, overweight, obesity, and inflammation, increased oxidative stress i is considered to be a major contributing factor to the increased incidence of certain CVDs (Senoner and Dichtl, 2019; Shaito et al., 2022). Oxidative stress refers to the excessive generation or accumulation of free radical species, such as the oxygen reactive species (ROS) (Di Meo and Venditti, 2020). Previous studies have shown that paeoniflorin can not only downregulate ROS-producing systems, but also intensifying antioxidant enzyme systems, which helps in managing ROS levels and ameliorating the pathological damage induced by oxidative stress (Han et al., 2022; Hu and Yang, 2022; Mao et al., 2022). For instance, paeoniflorin can increase superoxide dismutase (SOD) levels, reduce malondialdehyde (MDA) concentration, and upregulate nuclear factor erythroid factor 2-related factor 2 (Nrf2) expression, thereby augmenting liver antioxidant capacity and protecting the liver from oxidative stress (Hu et al., 2017). At present, At the moment, there is only a limited number of studies regarding the antioxidant effects of paeoniflorin on CVDs. Elevated ROS levels can induce inflammation and mitochondrial dysfunction, thereby affecting endothelial cell and macrophage function and ultimately accelerating the occurrence of CVDs (Forstermann et al., 2017). Paeoniflorin can suppress the secretion of cytokines and the expression of cyclooxygenase-2 (COX-2) and iNOS in a dose-dependent manner, at the same time diminish ROS accumulation in cells without experiencing any effect on macrophage phagocytosis (Li et al., 2022). ROS can be generated in cells via the NADPH oxidase system, which consists of multiple membrane-associated and cytosolic components (Vignais, 2002). NADPH oxidase 2 (Nox2) and Nox4 are highly expressed in endothelial cells and play a role in endothelial cell-cell adhesion and motility, which represent crucial elements in the oxidative stress-induced arteriosclerosis (Van Buul et al., 2005). Paeoniflorin can reduce ROS production by inhibiting the ROS-NF-κB axis and reducing Nox2/Nox4 expression, resulting in downregulation of HIF-1alpha/VEGF levels, alleviation of mitochondrial dysfunction, and protection of human umbilical vein endothelial cells from oxidative damage induced by AOPP (Song et al., 2017). Furthermore, paeoniflorin is capable of diminishing ROS levels in cardiomyocytes by downregulating the expression of microRNA-1, thereby improving cardiomyocyte viability and restraining cardiomyocyte apoptosis induced by doxorubicin (a highly potent anthracycline antitumor antibiotic) (Li et al., 2016) (Table 1).

## 4 Roles of paeoniflorin in various models of cardiac diseases

### 4.1 Anti-atherosclerotic effect

ASCVD is defined as an unequivocally diagnosed arteriosclerosis disease that includes acute coronary syndrome, stable coronary artery disease, post-revascularization, ischemic cardiomyopathy, ischemic stroke, transient cerebral ischemia, and peripheral arteriosclerosis disease. Factors such as dyslipidemia, impaired insulin sensitivity, inflammatory state, intense oxidative stress, endothelial dysfunction and other related factors may contribute to the initiation and progression of arteriosclerosis processes (Hill et al., 2021; Pirillo et al., 2021). Most ASCVD events can be avoided by preventing the formation of risk factors and by controlling traditional cardiovascular factors (Arnett et al., 2019). In case of ASCVD, adequate drug treatment is exceptionally critical to impede the progression of the disorder, and a combination of drugs instead of augmenting the amount of a single medication can create greater efficacy and decrease risks (Kim et al., 2022). Paeoniflorin, having multiple pharmacological actions, has great utilization potentiality in managing ASCVD. The regulatory effects of paeoniflorin on glucose and lipid metabolism, inflammation, and oxidative stress suggest its remarkable anti-arteriosclerotic characteristics, as is documented in 3.1–3.4. Equally, paeoniflorin can improve the pathological morphology of the aorta in atherosclerotic rats and alleviate atherosclerosis-related inflammation by inhibiting the TLR4/MyD88/NF-κB pathway (Li et al., 2017a). The underlying pathological mechanism of arteriosclerosis involves the proliferation, migration and inflammatory response of vascular smooth muscle cells (VSMCs) (Bennett et al., 2016). It was found that paeoniflorin could activate HO-1, induce cell cycle arrest, inhibit the p38/ERK1/2/MAPK/NF-κB signaling pathway, inhibit VSMC proliferation and migration induced by ox-LDL in a dose-dependent manner, and reduce the expression of inflammatory cytokines and chemokines (Li et al., 2018a). Paeoniflorin can also promote VSMC apoptosis by upregulating caspases (Guo et al., 2017). Furthermore, paeoniflorin made distinctive anti-platelet effects, counteracting platelet aggregation and clotting and significantly deterring intra-arterial thrombosis (Koo et al., 2010; Xie et al., 2017; Ngo et al., 2019). However, there is a lack of relevant mechanistic studies, so *in vivo* or *in vitro* experiments should be designed to investigate the specific mechanism behind paeoniflorin’s anticoagulant and anti-platelet aggregation effects (Table 2).

**TABLE 2:** Roles of paeoniflorin (PF) in various models of cardiac diseases.

Disease	Type	Model	Dose (PF)	Group	Duration	Mechanism	References
AS	Vitro	Ox-LDL-induced VSMCs	20, 40, 80 μM	(1) Control; (2) Ox-LDL-induced VSMCs (100 μg/mL); (3) Ox-LDL + PF group (PF: 20, 40, 80 μM).	24 h	PF inhibits VSMCs proliferation and migration by arresting cell cycle and activating HO-1 through MAPKs and NF-κB pathway.	Li et al. (2018a)
AS	Vitro	Non-alcoholic fatty liver disease model (HFD-fed C57BL/6J mice)	25, 50, 100 μg/mL	(1) Control; (2) Low-PF group (25 μg/mL); (3) Medium-PF group (50 μg/mL); (4) High-PF group (100 μg/mL).	12, 24, 48 h	PF inhibits VSMCs proliferation by down-regulating proteins associated with the nuclear factor-κB signaling pathway and promotes VSMCs apoptosis by up-regulating the expression of cystathione aspartase.	Guo et al. (2017)
Cardiac dysfunction	Vivo	LPS-induced cardiac dysfunction in C57BL/6 mice	15 mg/kg	(1) Control; (2) PF group (15 mg/kg; sterile saline dissolved with 0.5% Tween 80); (3) LPS group (10 mg/kg, sterile saline dissolved); (4) LPS + PF group.	3 days	PF attenuates cardiac dysfunction in endotoxemic mice via the inhibition of NF-κB pathway.	Tomek and Bub (2017)
Cardiac remodeling	Vivo	Cardiac remodeling in spontaneous hypertensive rats (SHR)	2.25, 4.50, 9.00 mg/kg	(1) Control (Wistar-Kyoto rats); (2) SHR group; (3) Low-PF treatment (2.25 mg/kg/d); (4) Medium-PF treatment (4.50 mg/kg/d); (5) High-PF treatment (9.00 mg/kg/d); (6) Captopril treatment (13.5 mg/kg/d).	8 weeks	PF improves pressure overload-induced cardiac remodeling by modulating the MAPK signaling pathway.	Liu et al. (2019a)
Cardiac remodeling	Vivo	Pressure overload-induced cardiac remodeling	20 mg/kg	(1) Sham operated control (saline); (2) Sham + PF (20 mg/kg); (3) AB (saline); (4) AB+ PF.	7 weeks	PF attenuates pressure overload-induced cardiac remodeling via inhibition of TGFBβ/Smads and NF-κB pathways.	Zhou et al. (2013)
Acute myocardial infarction	Vivo	Ventricular remodeling in AMI rats	2.25, 4.50, 9.00 mg/kg	(1) Sham operated control; (2) Model control; (3) Captopril group (4.50 mg/kg/d); (4) Low-PF treatment (2.25 mg/kg/d); (5) Medium-PF treatment (4.50 mg/kg/d); (6) High-PF treatmen (9.00 mg/kg/d).	28 days	PF decreases BNP, TNF-α and IL-6 levels, increases IL-10 levels and further inhibits the expression of cystathionin-3 and cystathionin-9.	Chen et al. (2018b)
Acute myocardial infarction	Vivo	Myocardial ischemic damage in AMI rats	5, 10, 20 mg/kg	(1) Sham operated control; (2) Vehicle group (saline + AMI); (3) PF treatment groups (AMI + PF 5, 10, 20 mg/kg).	7 days	PF ameliorates acute myocardial infarction of rats by inhibiting inflammation and inducible nitric oxide synthase signaling pathways.	Chen et al. (2015)
I/R	Vivo	Myocardial I/R induced injury in Sprague-Dawley rats	10 mg/kg	(1) Sham rats underwent surgical operation, but without occlu-sion of LAD; (2) Ischemia (25 min) and subsequent reperfusion (24 h) and the treatment with placebo (saline 0.3 mL); (3) Pretreatment with PF (10 mg/kg) before I/R injury.	1 h	PF can reduce myocardial damage in rat through protection from apoptosis.	Nizamutdinova et al. (2008)
I/R	Vivo	Myocardial I/R model	15, 30, 60 mg/kg	(1) Sham group; (2) Model group; (3) Low-PF group (15 mg/kg); (4) Medium-PF group (30 mg/kg); (5) High-PF group (60 mg/kg).	7 days	PF can reduce oxidative stress and apoptosis by inhibiting the expression of apoptosis-related signaling pathway.	Wu et al. (2020)
Hypertension	vitro	Blocking effect of compounds on calcium channels by live-cell imaging analysis (HEK 293 and H9C2)	Moutan Cortex: 1, 0.1, 0.01 mg/mL	(1) Control group; (2) Model group; (3) nifedipine group (10–5 mol/L); (4–6) Three Moutan Cortex groups with different concentrations (1, 0.1, 0.01 mg/mL).	6 h	PF can effectively block voltage-operated Ca^2+^ channels (VOCCs) to exert calcium antagonism.	Lu et al. (2019)
Arrhythmic	vitro	Isolated rat ventricular myocytes or transfected human embryonic kidney 293 (HEK293) cells	10 mmol/L	(1) control; (2) PF group (10 mmol/L).	2 h	PF can block I(Ca-L), I(Na), and I(K1) without affecting I(to1), I(Ks), or I(Kr).	Wang et al. (2011)
Angiogenesis	Vivo + vitro	Vivo: A vascular insufficiency model in the Tg(fli-1:EGFP)y1 transgenic zebrafish	Vivo: 6.25–100 μmol/L	Vivo: (1) vehicle control (embryo water containing 0.1% DMSO); (2) VRI group (300 ng/mL) (2) VRI + PF group (6.25, 12.5, 25, 50, 100 μmol/L);	Vivo: 24 h	The mechanism of PF pro-angiogenic action may be related to the activation of VEGF signaling pathway.	Xin et al. (2018)
		Vitro: HUVECs	Vitro: 0.001, 0.003, 0.01, 0.03, μmol/L; 0.3, 1, 10 μmol/L; 0.3–10 μmol/L	vitro: (1) Vehicle control (DMSO 0.1%); (2) Positive control (VEGF 20 ng/mL); (3) PF group.	vitro: MTT assay—24 h; wound healing assay - 10 h; tube formation assay—4 h		
Vascular remodeling	vitro	PDGF-BB - induced proliferation of primary cultured rat VSMCs	50, 100, 200 μM	(1) Control; (2) PDGF-BB group (1 ng/mL); (3) PDGF-BB + PF group (50, 100, 200 μM); (4) PF group.	Flow cytometry analysis of cell cycle progression—20 h; Scratch migration test—24 h; ROS measurement—1 h;	PF suppresses PDGF-BB-induced VSMC proliferation through the ROS-mediated ERK1/2 and p38 signaling pathways.	Fan et al. (2018)
Angiogenesis	vitro	Angiogenesis in ox-LDL-induced HUVECs	10, 1, 0.1, 0.01 μmol/L	(1) Control; (2) Ox-LDL group (20 μg/mL); (3) Ox-LDL + PF group (10, 1, 0.1, 0.01 μmol/L).	24 h	PF suppresses ox-LDL-induced angiogenesis in HUVECs by inhibiting both the VEGF/VEGFR2 and the Jagged1/Notch1 signaling pathways.	Yuan et al. (2018)
Insulin resistance	vitro	Human HepG2 cells	3, 30, 100 mM	(1) Control; (2) PA group (0.25 mM); (3) PA + PF group (3, 30, 100 mM).	1 h	PF suppresses lipid accumulation and alleviates insulin resistance by regulating the Rho kinase/IRS-1 pathway in palmitate-induced HepG2Cells.	Ma et al. (2017b)
Insulin resistance	vivo	Fructose-induced insulin resistance and hepatic steatosis in Sprague-Dawley rats	10, 20, 40 mg/kg	(1) Control group (Saline); (2) Fructose group (20% Fructose drink); (3–5) Fructose + PF group (10, 20, 40 mg/kg); (6) Fructose + pioglitazone group (10 mg/kg).	8 weeks	PF ameliorates fructose-induced insulin resistance and hepatic steatosis by activating LKB1/AMPK and AKT pathways.	Li et al. (2018c)
Diabetes	vitro	Rat insulin-secreting beta-cell line (INS-1)	20, 40, 80 μM	(1) Control; (2) STZ group (3 mmol/L); (3) STZ + PF group (20, 40, 80 μM).	2 h	PF protects pancreatic beta cells from STZ-induced damage through inhibition of the p38 MAPK and JNK signaling pathways.	Liu et al. (2019c)

Abbreviations**:** AB, aortic banding; AMI, acute myocardial infarction; AS, atherosclerosis; HEK 293, human embryonic kidney cells; HUVECs, human umbilical vein endothelial cells; H9C2, rat myocardial cells; SHR, spontaneous hypertensive rats; VSMCs, vascular smooth muscle cells; MAPK, mitogen-activated protein kinase; NF-κB, nuclear factor-kappa B; PDGF-BB, platelet derived growth factor-BB; STZ, streptozotocin; VRI, VEGF receptor tyrosine kinase inhibitor II.

### 4.2 Improvement of cardiac function and inhibition of cardiac remodeling

Many studies have shown that paeoniflorin has pharmacological effects which improve cardiac function and inhibit cardiac remodeling. Paeoniflorin ameliorates cardiac dysfunction and regulates the levels of inflammatory cytokines (e.g., IL-1β, IL-6, IL-12, MCP-1, IFN-γ, and iNOS), by affecting the PI3K/AKT signaling pathway and reducing inflammation-related damage (Zhai and Guo, 2016). What is more, paeoniflorin has been proven to be effective in inhibiting cardiac remodeling and alleviating myocardial infarction and ischemia-reperfusion injury. It should be noted that cardiac remodeling denotes an alteration in the size, shape, and function of the heart, which is triggered by gene expression changes induced by cardiac injury or hemodynamic stress and is drastically correlated to hypertension as well as other cardiovascular abnormalities (Tomek and Bub, 2017). Of all these alterations, myocardial hypertrophy and fibrosis are the most predominant, and the severity of myocardial hypertrophy and fibrosis is closely related to the mortality of patients following heart failure (Heinzel et al., 2015; Shenasa and Shenasa, 2017). Paeoniflorin attenuates cardiac hypertrophy, fibrosis, and inflammation in spontaneously hypertensive rats by inhibiting MAPK signaling, and ameliorates pressure overload-induced cardiac remodelling (Liu et al., 2019a). Moreover, to validate the efficacy of paeoniflorin, a model of cardiac remodeling was established by aortic band (AB)-induced pressure overload in mice. The findings demonstrated that treatment with paeoniflorin decreased the heart weight to body weight ratio (HW/BW), reduced the expression of hypertrophic genes, inhibited the apoptosis of cardiomyocytes, alleviated myocardial fibrosis and improved ventricular function by suppressing the activity of the TGF-β/Smads/NF-κB pathways (Zhou et al., 2013). Strikingly, another study also demonstrated that paeoniflorin inhibited the TGF-β1/Smad signaling pathway to reduce cardiac remodeling in an isoproterenol (Iso)-induced rat cardiac remodeling model (Liu et al., 2019b) (Table 2).

Ischemic heart disease, myocardial infarction (MI), hypertension, and valvular heart disease (VHD) are common causes of heart failure (Heidenreich et al., 2022). In China, the occurrence and development of ventricular remodeling after myocardial infarction are the main causes of heart failure. To test this, a rat model of acute myocardial infarction (AMI) was established by ligation of the anterior descending coronary artery, and paeoniflorin was administered orally for 4 weeks post-surgery. Subsequent examination of doppler ultrasonography showed significantly increased left ventricular ejection fraction (LVEF), decreased left ventricular end-diastolic diameter (LVIDd), and decreased left ventricular end-systolic diameter (LVIDs). Furthermore, pathological results from myocardial samples (pericardium tissue) taken from within a 2 mm radius from the visible edge of the infarct showed that paeoniflorin treatment decreased myocardial degeneration in rats. Collectively, these results highlight the ability of paeoniflorin to enhance cardiac function and mitigate the adverse remodeling of the left ventricle post-infarction (Chen et al., 2018b). Additionally, an additional study attributed paeoniflorin’s cardioprotective effects to its ability to reduce inflammation and inhibit the iNOS signaling pathway (Chen et al., 2015) (Table 2).

### 4.3 Alleviation of ischemia-reperfusion injury

Early access to coronary intervention following myocardial infarction is a crucial measure for improving prognosis, yet it is paramount to not underestimate ischemia-reperfusion (I/R) injury that may occur after revascularization (Yellon and Hausenloy, 2007). To assess the effects of Paeoniflorin on I/R injury, a rat study was conducted, in which 10 mg per kilogram (mg/kg) of Paeoniflorin was intraperitoneally injected 1 h before I/R injury. The results of this study demonstrated that Paeoniflorin significantly improved hemodynamic parameters and reduced myocardial infarction size, as well as downregulated the expression of caspase-3 and Bax, while upregulating the expression of Bcl-2, thus indicating that Paeoniflorin can protect against I/R injury through its anti-apoptotic action (Misao et al., 1996). Another study also revealed the potential of Paeoniflorin to have immediate effects on I/R injury (Nizamutdinova et al., 2008). As the occurrence times of acute cardiac events cannot be predicted during clinical diagnosis and treatment, a course of traditional Chinese medicine preparations are usually taken orally for a certain period of time in order for its therapeutic properties to be adequatelyeffective. Prior to constructing the I/R rat model, Wu et al. orally administered Paeoniflorin for seven consecutive days to the experimental animals, with the last dose being 30 min prior to the induction of ischemia (Wu et al., 2020). It was shown that paeoniflorin pretreatment significantly reduced the size of myocardial infarction, the degree of myocardial injury, apoptosis, and oxidative stress. The specific mechanism of action may be related to the regulation of the MAPK signaling pathway (Wu et al., 2020) (Table 2).

### 4.4 Improvement of hypertension and arrhythmia

Paeoniflorin also has the pharmacological effect of improving hypertension and arrhythmia. Studies have found that, compared with its single use, the combination of Paeoniflorin-enriched extract and metoprolol can enhance the bioavailability of Paeoniflorin and contribute to a greater anti-hypertensive effect; it can concurrently reduce systolic and diastolic blood pressure in spontaneously hypertensive rats (SHR), increase NOS expression in vascular endothelium, improve the arrangement of elastic fibers and cell hypertrophy in the vascular wall, as well as limit aortic vascular damage and other organ damages (Li et al., 2017b; Li et al., 2018b). Yu et al. suggested that the anti-hypertensive mechanism of paeoniflorin may be related to the effective blocking of voltage-controlled calcium channels (VOCCs) (Lu et al., 2019). This hypothesis is further supported by research which suggests that Paeoniflorin not only blocks L-type calcium current (I(Ca-L)), inward rectifier potassium current (I(K1)) and sodium current (I(Na)) in rat cardiomyocytes, but does so without affecting the instantaneous outward potassium current (I(to1)), slow delayed rectifier current (I(Ks)) and HERG current (I(Kr)), which may partly explain its anti-arrhythmic effects with minimal pro-arrhythmic potential (Wang et al., 2011) (Table 2).

### 4.5 Regulation of angiogenesis

Angiogenesis has been shown to be beneficial in patients with cardiac insufficiency or myocardial infarction (Oka et al., 2014). Paeoniflorin has been demonstrated to bidirectionally regulate angiogenesis, and its angiogenic effect has been demonstrated in a zebrafish model of vascular insufficiency, as well as in human umbilical vein endothelial cells (HUVECs) (Xin et al., 2018). When Platelet-derived growth factor BB (PDGF-BB) is released in the context of vascular injury, it binds to the cell membrane receptor PDGFR-β and activates NADPH oxidase to generate large amounts of ROS. This leads to aberrant proliferation and migration of vascular smooth muscle cells (VSMCs), and consequently to arteriosclerosis and restenosis (Rivard and Andres, 2000; Lee et al., 2007; Ding et al., 2015). In such cases, it is important to limit angiogenesis. Paeoniflorin has been shown to inhibit PDGF-BB-induced VSMC proliferation by modulating ERK12 and p38 signaling pathways, suggesting the potential of paeoniflorin to act as a therapeutic for arteriosclerosis and restenosis following a percutaneous coronary intervention (Fan et al., 2018). In addition, paeoniflorin can stabilize arteriosclerosis plaques, by inhibiting both the VEGF/VEGFR2 and Jagged1/Notch1 signaling pathways (Yuan et al., 2018) (Table 2).

### 4.6 Improvement of insulin resistance

The contribution of insulin resistance and diabetes to the pathogenesis of CVDs has already been established (Fox et al., 2007; Hill et al., 2021). CVDs is considered to be the leading cause of death and complications in type 1 diabetes (T1D) mellitus and type 2 diabetes (T2D) mellitus (Cheng et al., 2018). The main pathological feature of T2D mellitus is chronic insulin resistance (Madsbad, 1992). The gradual decline in pancreatic β cell function is the main cause of impaired insulin sensitivity (Shoemaker et al., 2015). Recent studies have shown that paeoniflorin can improve insulin resistance and protect β cells, by inhibiting the activation of Rho kinase (ROCK) and serine phosphorylation of INSR substrate (IRS)-1, and promoting AKT and glycogen synthase kinase (GSK)-3β phosphorylation (Ma et al., 2017b). Additionally, paeoniflorin can also reduce serum insulin and glucagon levels and improve insulin sensitivity by activating the LKB1/AMPK signaling pathway (Li et al., 2018c). Furthermore, paeoniflorin also can significantly ameliorate pancreatic beta cell injury, and regulate glucose metabolism by inhibiting the p38 MAPK and JNK signaling pathways (Liu et al., 2019c) (Table 2).

## 5 Pharmacokinetics of paeoniflorin

Since 1985, research on paeoniflorin pharmacokinetics has gradually deepened and its effects become better understood (Hattori et al., 1985; Shen et al., 2021). Researchers have delved into studying of the processes of absorption, distribution, biochemical conversion (or metabolism), and excretion of paeoniflorin in the body, particularly its changes in blood concentration over time, which is of great help for the further development and application of paeoniflorin. The usual bioavailability of paeoniflorin absorbed by oral or intestinal perfusion is approximately at 2%–4% (Fei et al., 2016; Wang et al., 2016; Yu et al., 2019). Poor fat solubility of paeoniflorin, P-glycoprotein (P-gp)-mediated transport mechanism, and degradation by gut microbiota enzymes are causes that impede its bioavailability (Liu et al., 2006; Yu et al., 2019).

After absorption, paeoniflorin is widely distributed in various tissues, such as the heart, liver, spleen, lung, kidney, stomach, and intestines (Luo et al., 2014). Additionally, paeoniflorin can pass the blood-brain barrier by passive diffusion (Luo et al., 2014; Hu et al., 2016). Many researchers think it is the metabolite of paeoniflorin, benzoic acid, that actually reaches the barrier mentioned above (Yu et al., 2019). Most paeoniflorin is mainly eliminated in the urine via glomerular filtration of the renal system (Cheng et al., 2016). As experimental data collected from rats show, the serum concentration of paeoniflorin is dose-dependent, with a half-life (T1/2) of approximately 1.8 h (Fei et al., 2016; Chen et al., 2021). In traditional Chinese medicine, Chinese herbal preparations made by combinations of various kinds of botanical drugs can usually achieve the role of synergistic effect and attenuation of toxicity. Moreover, studies show that the compatibility of such botanical drugs or the combination of their active ingredients can play a role in altering paeoniflorin’s pharmacokinetic parameters: when combined with Angelica sinensis, its absorption rate is accelerated, peak time (T max) is shortened, T1/2 is increased, mean residence time (MRT) is prolonged, and its distribution within said tissues is widened (Luo et al., 2014). Conversely, when combined with glycyrrhizic acid, it has been noticed that the T1/2 of paeoniflorin reduces, while its drug clearance and metabolism in rats speed up (Sun et al., 2019). In addition, paeoniflorin displays distinct effects when used in combination with other pharmaceuticals such as quinidine, verapamil, sinomenine, and cyclosporine A (Chan et al., 2006; Liu et al., 2006; Gong et al., 2015; Xu et al., 2016; He et al., 2017).

## 6 Clinical application

**FIGURE 3:**
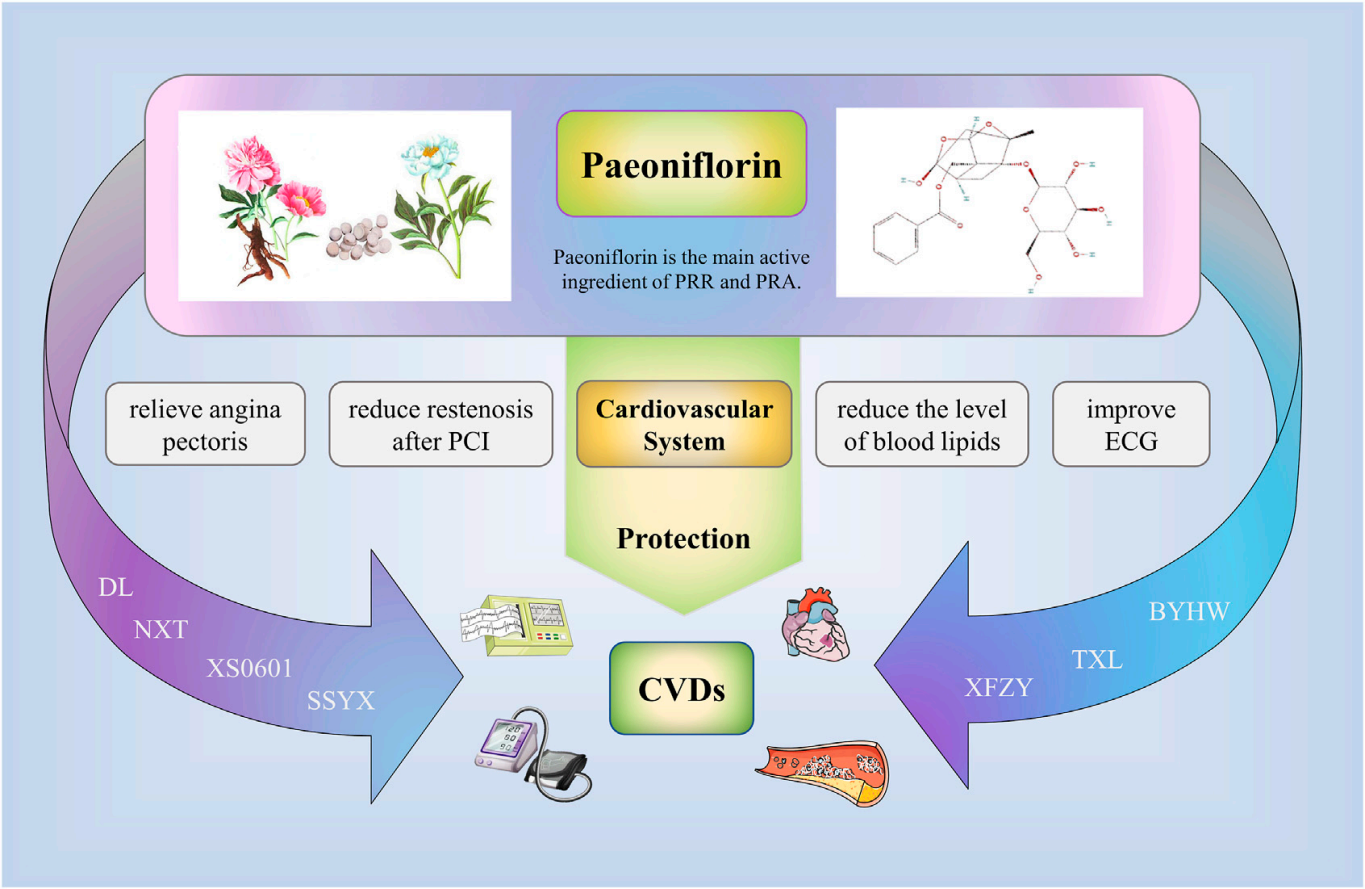
Clinical application of paeoniflorin. Some Chinese patent medicines or preparations containing Peony or paeoniflorin showed good cardiovascular protection; for example, XS0601, Naoxintong (NXT), Buyang Huanwu (BYHW), Xuefu Zhuyu (XFZY), Shensong Yangxin (SSYX), Tongxinluo (TXL), Danlou (DL). The above drugs can reduce the symptoms of angina pectoris, reduce the restenosis after PCI, reduce the level of blood lipids, improve ECG, and effectively treat the related cardiovascular diseases.

At present, the clinical application of paeoniflorin in traditional Chinese medicine is seldom studied by searching the relevant databases (Figure 3). Peony is the main source of paeoniflorin, especially Chishao (Zhang et al., 2022). Some Chinese patent medicines or preparations containing Peony or paeoniflorin showed good cardiovascular protection; for instance, XS0601 consists of active ingredients (Paeoniflorin and Chuangxiongol) that have been shown through animal studies to inhibit neointimal hyperplasia arteries (Xu et al., 2001). Notably, a multicenter, randomized, double-blind, placebo-controlled trial involving 335 patients has confirmed that administering of XS0601 for 6 months significantly reduces restenosis after percutaneous coronary intervention (PCI) (Chen et al., 2006). Naoxintong is composed of Chishao and 15 additional botanical drugs (Han et al., 2019). It has been proved through clinical trials to protect endothelial cells and treat coronary artery disease (Lv et al., 2016; Long-Tao, 2018). Supplementing aspirin with NXT has further been revealed to heighten the antiplatelet effect in cerebrovascular disease patients (Chen et al., 2008a). Buyang Huanwu (BYHW) decoction containing Chishao is also a traditional Chinese medicine compound preparation. Relevant clinical studies and meta-analysis have proved that its therapeutic effect on stable angina pectoris (SAP)and stroke (Zhang et al., 1995; Gao et al., 2021; Wang et al., 2022a). Recently, Wang et al. (2022b) designed a randomized, blinded, parallel controlled, multicenter clinical trial to compare the efficacy and safety of NXT and BYHW in the treatment of SAP. The results of the trial have yet to be published. Xuefu Zhuyu (XFZY) decoction is composed of Chishao and 10 additional botanical drugs (Li et al., 1998). It has great advantage in treating coronary heart disease. It can effectively relieve symptoms of angina pectoris, improve ECG, reduce the level of blood lipids, and improve endothelial function, among others (Wang and Qiu, 2019; Zhang et al., 2021b). Additionally, Shensong Yangxin (SSYX) capsule consists of Chishao and 11 additional botanical drugs. A randomized, double-blind, controlled, multicenter trial demonstrated a significant effect of SSYX capsules in reducing the number of premature ventricular contractions (PVC) and relieving symptoms associated with PVC compared with placebo or mexiletine (Zou et al., 2011). Furthermore, Tongxinluo (TXL) capsule contains radix paeoniae rubra and 11 additional botanical drugs (Hao et al., 2015). Related clinical studies have shown that TXL can anti arteriosclerosis, reduce blood lipid levels, improve angina pectoris, reduce the incidence of restenosis, and significantly reduce the incidence of no-reflow and myocardial infarction area after primary PCI (Chen et al., 2008b; Chen et al., 2011; Zhang et al., 2019). Danlou (DL) tablets are also a kind of Chinese patent medicine composed of Chishao and other botanical drugs. It has been widely used to treat coronary artery disease in China for a long time. Relevant clinical research demonstrated that DL can treat stable angina pectoris, alleviate adverse left ventricular remodeling after myocardial infarction, and reduce the peri-procedural myocardial injury among patients undergoing PCI for non-ST elevation acute coronary syndrome (Wang et al., 2015; Mao et al., 2016; Yang et al., 2020; Zhao et al., 2021).

## 7 Conclusion and future directions

**FIGURE 4:**
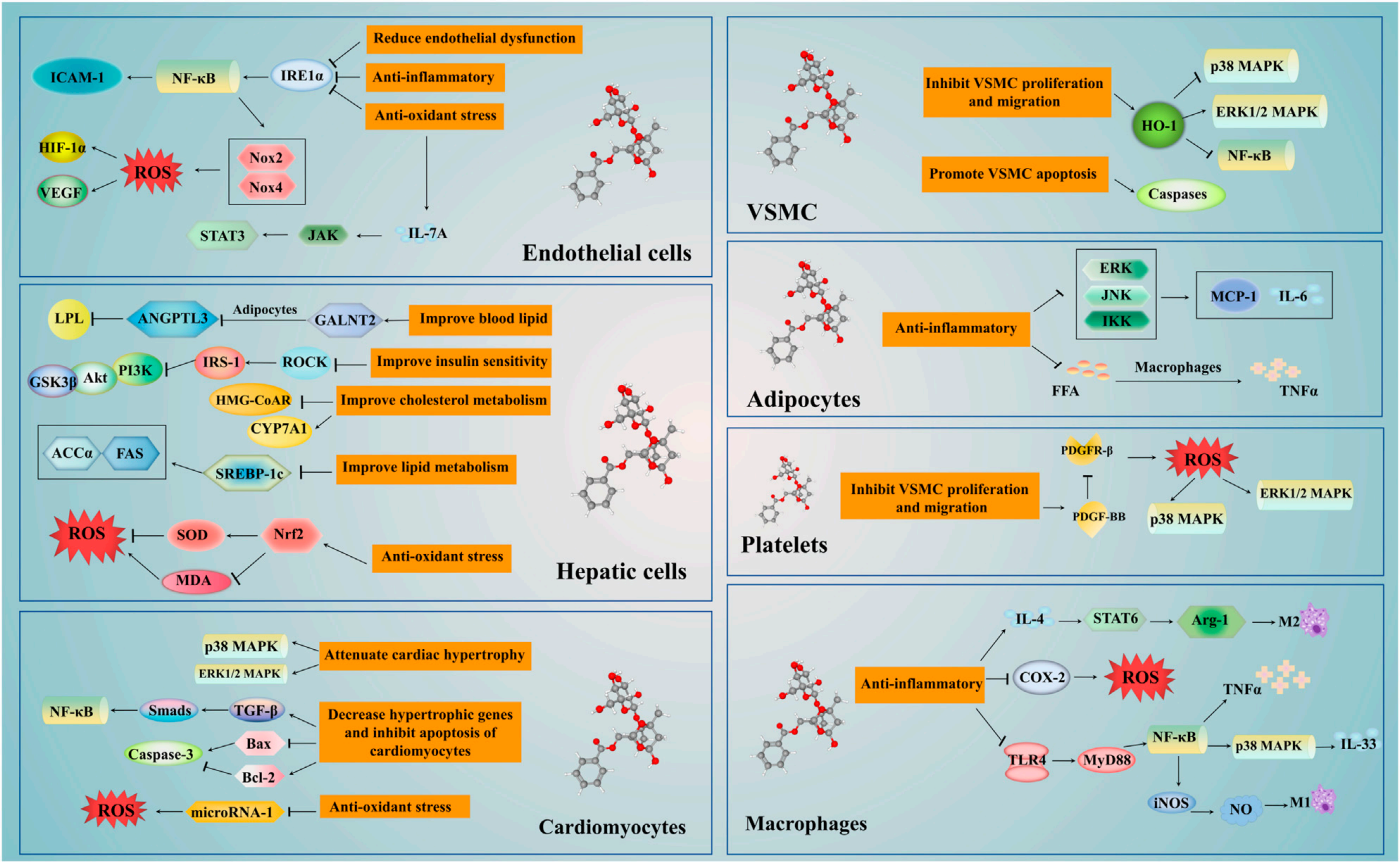
The mechanism of Paeoniflorin in the treatment of cardiovascular disease. The treatment of paeoniflorin has the advantage of multi-targets and multi-pathways. It regulates the NF-κB, p38 MAPK, ERK 1/2 MAPK and other signaling pathways in the liver, macrophages, adipocytes, vascular endothelial cells, smooth muscle cells and myocardial cells. Paeoniflorin can regulate insulin sensitivity and liver activity, and improve glucose and lipid metabolism. Moreover, paeoniflorin can improve the proliferation and migration of VSMCs, regulate the levels of blood lipids, protect the vascular endothelium and achieve the goal of anti-atherosclerosis through exerting anti-inflammatory and antioxidant stress. In addition, paeoniflorin can inhibit cardiomyocyte apoptosis and myocardial remodeling, reduce ischemia-reperfusion damage, and improve cardiac function. Abbreviations: ANGPTL3, angiopoietin like protein 3; COX-2, cyclooxygenase-2; CYP7A1, cytochrome P4507A1; FFA, free fatty acids; GALNT-2, N-acetylgalactosaminyltransferase 2; GSK3β, glycogen synthase kinase (GSK)-3β; HMG-CoAR, 3-hydroxy-3-methylglutaryl coenzyme A reductase; ICAM-1, inter cellular adhesion molecule-1; IL, interleukin; iNOS, nitric oxide synthase; IREα, inositol enzyme 1alpha; IRS, INSR substrate; LPL, lipoprotein lipase; MCP-1, monocyte chemoattractant protein-1; MDA, malondialdehyde; NF-κB, ; Nrf2, nuclear factor erythroid factor 2-related factor 2; Nox, NADPH oxidase; PDGF-BB, platelet-derived growth factor BB; ROCK, Rho kinase; ROS, reactive oxygen species; SOD, superoxide dismutase; TGF-β, transforming growth factor-β; TLR, toll like receptors; TNF-α, tumor necrosis factor-α; VSMCs, vascular smooth muscle cells.

Paeoniflorin, an effective component of natural plants, protects the cardiovascular system through multiple pharmacological actions (Figure 4). It can regulate lipid synthesis and metabolism via numerous processes, such as the *de novo* synthesis, lipid oxidation, cholesterol synthesis, and output. Also, it can inhibit the inflammatory response induced by the NF-κB signaling pathway through multiple targets, regulate macrophage function, and inflammation-induced endothelial dysfunction. Additionally, paeoniflorin decreases oxidative stress-induced cellular dysfunction by decreasing the excessive production or accumulation of ROS. It can also stymie atherosclerosis by decreasing cholesterol deposition, and eradicating inflammatory, oxidative and platelet aggregation effects. Besides, it ameliorates cardiac dysfunction by regulating the PI3K/Akt signalling pathway, attenuating cardiac remodeling, and alleviating ischemia-reperfusion injury inspired by inhibition of the MAPK signaling pathway. Not only this, but paeoniflorin also has various pharmacological benefits, such as reduction in blood pressure, arrhythmia improvement, angiogenesis regulation, and lucubration of insulin resistance.

In summary, paeoniflorin is a natural drug with high potential development. However, pharmacokinetic studies have shown that its low bioavailability, and therefore necessitating combining it with other traditional Chinese medicines to significantly improve its pharmacokinetic parameters. It has been shown that the esterified derivatives of paeoniflorin could improve the bioavailability and have beneficial pharmacodynamics. Consequently, how to further develop paeoniflorin, improve its bioavailability and extend its medicinal applications is the next focus in pharmaceutics. In regards to safety, certain basic experiments have revealed hepatoprotective effects of paeoniflorin, such as its ability to interfere with bile acid metabolism and pivotal inflammation-related targets, as well as its capacity to ameliorate cholestatic liver injury (Wei et al., 2020; Liu et al., 2022). Despite this, whether paeoniflorin could cause liver injury has not been declared yet. In addition, paeoniflorin is mainly excreted in urine through glomerular filtration, and its effect on renal function has not been reported. In attempts to promote paeoniflorin application, it is essential to analyze its possible toxicity and safety.

At present, the protective effects of paeoniflorin on CVDs are mainly based on animal or cell experimental model. Most of the corresponding clinical studies mainly address Chinese herbal compound preparations containing paeoniflorin or Chishao, with popular treatments including NXT, BYHW, XFZY, Shensong Yangxin, TXL,DL, and so on. Although most studies have proved that paeoniflorin has a wide range of effects on the prevention and treatment of CVDs, but the above studies inevitably have some objective limitations, which is not enough for the scientific research and clinical application of paeoniflorin. Therefore, the next step is to carefully design multicenter, large-scale, and randomized controlled trial studies to assess the efficacy and toxicological characteristics of paeoniflorin alone for CVDs. In addition, paeoniflorin’s basic research, including metabonomics, proteomics, genomics and network pharmacology, should be carried out to fully understand its pharmacological effects and molecular mechanisms. As a therapeutic agent with significant medical application potential, paeoniflorin is worthy of further development and utilization in the foreseeable future.
